# Recombinant TSR1 of ADAMTS5 Suppresses Melanoma Growth in Mice via an Anti-angiogenic Mechanism

**DOI:** 10.3390/cancers10060192

**Published:** 2018-06-11

**Authors:** Bhuvanasundar Renganathan, Vinoth Durairaj, Dogan Can Kirman, Paa Kow A. Esubonteng, Swee Kim Ang, Ruowen Ge

**Affiliations:** Department of Biological Sciences, Faculty of Science, National University of Singapore, Singapore 117543, Singapore; dbsbr@nus.edu.sg (B.R.); dbsduv@nus.edu.sg (V.D.); cankirman@u.nus.edu (D.C.K.); paakowebch@yahoo.com (P.K.A.E.); ang.science@gmail.com (S.K.A.)

**Keywords:** ADAMTS5, TSR1, apoptosis, anti-angiogenic, anticancer

## Abstract

Inhibiting tumor angiogenesis is a well-established approach for anticancer therapeutic development. A Disintegrin-like and Metalloproteinase with ThromboSpondin Motifs 5 (ADAMTS5) is a secreted matrix metalloproteinase in the ADAMTS family that also functions as an anti-angiogenic/anti-tumorigenic molecule. Its anti-angiogenic/anti-tumorigenic function is independent from its proteinase activity, but requires its first thrombospondin type 1 repeat (TSR1). However, it is not known if recombinant TSR1 (rTSR1) can function as an anticancer therapeutic. In this report, we expressed and purified a 75-residue recombinant TSR1 polypeptide from *E. coli* and investigated its ability to function as an anticancer therapeutic in mice. We demonstrate that rTSR1 is present in the blood circulation as well as in the tumor tissue at 15 min post intraperitoneal injection. Intraperitoneal delivery of rTSR1 potently suppressed subcutaneous B16F10 melanoma growth as a single agent, accompanied by diminished tumor angiogenesis, increased apoptosis, and reduced cell proliferation in the tumor tissue. Consistently, rTSR1 dose-dependently induced the apoptosis of cultured human umbilical vein endothelial cells (HUVECs) in a caspase-dependent manner. This work indicates that rTSR1 of ADAMTS5 can function as a potent anticancer therapy in mice. It thus has the potential to be further developed into an anticancer drug.

## 1. Introduction

The tumor microenvironment predominantly displays excessive angiogenesis, due to the high level of pro-angiogenic factors present [1]. Angiogenesis is needed not only for the sustained growth of a solid tumor, but also for tumor metastasis [2]. Factors inhibiting angiogenesis have been actively pursued as anticancer therapeutics. Amongst them, endogenous angiogenesis inhibitor proteins have gained much attention owing to their high specificity and low side effects [3,4]. 

A Disintegrin-like and Metalloproteinase with ThromboSpondin Motifs 5 (ADAMTS5) is an extracellular proteoglycanase of the ADAMTS family. It is widely studied for its role in osteoarthritis, as the main aggrecanase that cleaves aggrecans in the articular cartilage [5,6]. However, contrasting roles for ADAMTS5 have been reported with regards to cancer. While several studies have reported reduced expression of ADAMTS5 in cancers, such as prostate cancer [7], breast cancer [8], colorectal cancer [9], and hepatocellular carcinoma (HCC) [10], others have reported elevated expression of ADAMTS5 in human glioblastomas [11]. A recent report indicated that high ADAMTS5 expression in colorectal cancer patients correlated with higher levels of lymphatic invasion and lymph node metastasis [12]. Nevertheless, no significant differences in the overall survival and disease-free survival of these colorectal patients were observed. 

We have previously demonstrated that ADAMTS5 functions as an inhibitor of angiogenesis and suppresses xenograft tumor growth in mice [13]. We showed that this anticancer function is independent of the catalytic activity of ADAMTS5, but is mediated by its central ThromboSpondin type 1 repeat domain (TSR1). Stable overexpression of the TSR1 domain alone in B16 melanoma cells suppressed subcutaneous tumor growth in syngeneic mice. We also reported that *E. coli* produced recombinant TSR1 (rTSR1), but not the C-terminus TSR2 (rTSR2), which inhibits human umbilical vein endothelial cell (HUVEC) tube formation and proliferation in vitro. Additionally, rTSR1 triggers HUVEC apoptosis in the presence of vascular endothelial growth factor (VEGF) [14]. These findings suggest that rTSR1 polypeptide may have the potential to be developed as an anticancer therapeutic. 

In this study, we investigated whether rTSR1 can function as an anticancer therapeutic in a mouse xenograft model. We show that intra-peritoneal delivery of rTSR1 potently suppressed subcutaneous B16 melanoma growth in mice. This result establishes that rTSR1, a 75-residue polypeptide from ADAMTS5, warrants further investigation. It has the potential to be developed as an anticancer therapy for melanoma. 

## 2. Results

### 2.1. Recombinant Type 1 Repeat Domain Induces Caspase-Dependent Apoptosis in Human Umbilical Vein Endothelial Cells

**Figure 1:**
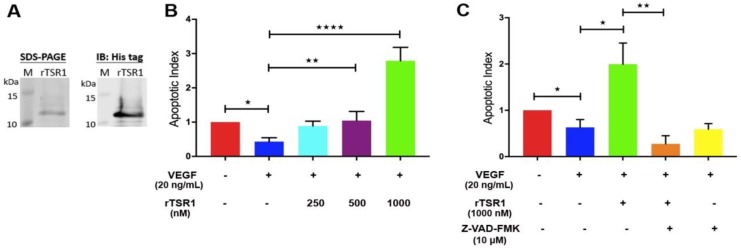
Recombinant type 1 repeat domain (rTSR1) induces caspase-dependent apoptosis in human umbilical vein endothelial cells (HUVECs). (**A**) SDS-PAGE and immunoblot analysis of purified rTSR1 showed a band at ~10 kDa; (**B**) rTSR1 dose-dependently induced apoptosis in HUVECs in the presence of VEGF. Data shown are apoptosis observed at 24 h post-1000 nM rTSR1 treatment; (**C**) Pan-caspase inhibitor (Z-VAD-FMK) significantly reduced the 1000 nM rTSR1-induced apoptosis at 24 h post treatment. Data represent the mean of triplicates ± SD. Statistical analysis performed by one-way ANOVA. * *p* < 0.05, ** *p* < 0.01, **** *p* < 0.0001.

We expressed the His-tagged 75-residue TSR1 domain of ADAMTS5 in *E. coli*. With the Ni-NTA (Nickel–Nitrilotriacetic acid) column, the expressed rTSR1 was purified as a single band at ~10 kDa in an SDS-PAGE and anti-His immunoblot (Figure 1A). No significant endotoxin was present in the purified rTSR1 (data not shown). Consistent with our previous report, rTSR1 showed significant pro-apoptotic activity at 500 nM and 1000 nM after 24 h incubation in the presence of VEGF (Figure 1B) [14]. Since prominent pro-apoptotic activity was observed at 1000 nM, this concentration was then used for subsequent experiments. The rTSR1 triggered HUVEC apoptosis in a caspase-dependent manner, and the presence of the pan-caspase inhibitor Z-VAD-FMK eliminated rTSR1 (1000 nM)-induced apoptosis (Figure 1C). 

### 2.2. Recombinant Type 1 Repeat Domain Suppressed the B16F10 Melanoma Growth in Mice

**Figure 2:**
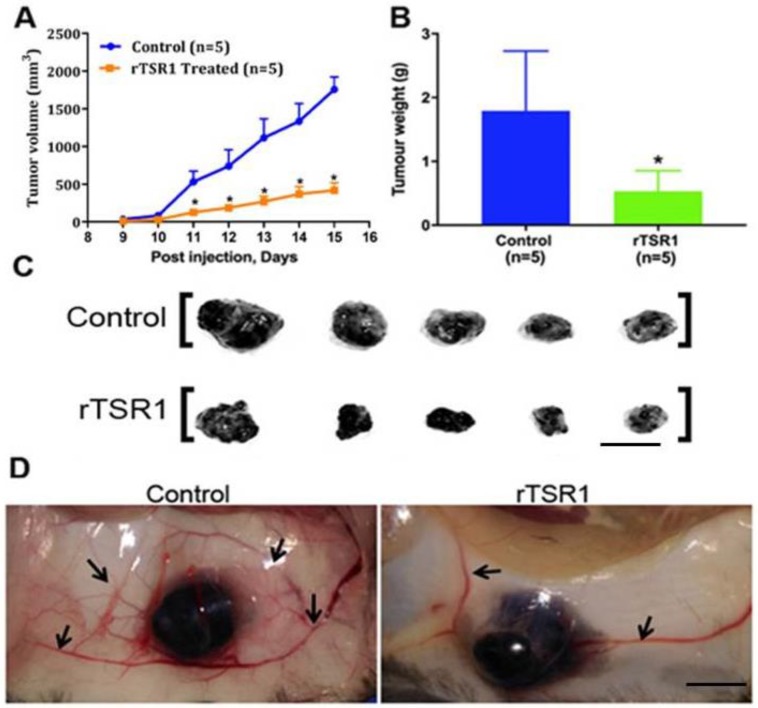
Systemically delivered rTSR1 suppressed B16F10 tumor growth in mice. (**A**) Tumor growth curve from day 9 to 15 post tumor cell injection (*n* = 5). Data represents mean ± SD. The rTSR1 was systemically delivered through intra-peritoneal (IP) injection. Statistical analysis performed by Student’s *t* test. * *p* < 0.05; (**B**) Tumor weight at the end of experiment, control and rTSR1 treated group, *n* = 5 for each group. The graph represents the mean ± SD of the tumor weight. Statistical analysis performed by Student’s *t* test. * *p* < 0.05; (**C**) Dissected tumor nodules from control and rTSR1 treated groups—scale bar: 1 cm; (**D**) rTSR1-treated mice showed a reduced peri-tumor vascular network compared to tumors of similar sizes in the control group—scale bar: 0.5 cm. Arrows indicate the blood vessels surrounding the tumor.

To investigate if rTSR1 can be a potential anticancer drug, the effect of systemically delivered rTSR1 on tumor growth was investigated. A subcutaneous tumor was generated by injecting half a million B16 melanoma cells into the dorsal flank of mice. On the same day, buffer or 200 μg of rTSR1 was injected intraperitoneally (IP) to each mouse to the control group and rTSR1 treatment group, respectively. Subsequently, rTSR1 was delivered to each mouse on alternate days. Tumor nodules were visible on day seven post-cell inoculation. Treatment with rTSR1 led to a significant reduction of tumor growth when compared to the control mice (Figure 2A). At the end of the experiment (on day 15 post-cell inoculation), tumor nodules were harvested, and significantly reduced tumor weights were observed (Figure 2B,C). Notably, rTSR1-treated mice had an obviously reduced peri-tumor vasculature, consistent with the anti-angiogenic activity of rTSR1 (Figure 2D). These data demonstrate that rTSR1 can function as an anticancer therapy as a single agent in mice, likely by restricting angiogenesis. 

### 2.3. Recombinant Type 1 Repeat Domain Suppresses B16 Melaonma Growth by Inhibiting Tumor Angiogenesis and Tumor Cell Proliferation While Inducing Tumor Cell Apoptosis

**Figure 3:**
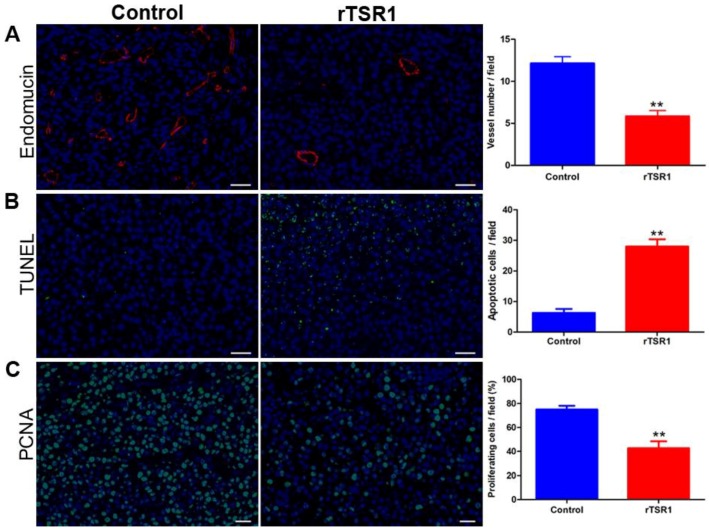
The rTSR1 suppressed angiogenesis and cell proliferation but induced apoptosis in tumors. Tumor paraffin sections were probed for microvessel density, tumor cell proliferation, and apoptosis through immunofluorescence staining, using (**A**) anti-endomucin, (**B**) TUNEL staining, and (**C**) anti-PCNA (Proliferating cell nuclear antigen) respectively. The corresponding quantification of the staining is presented in the bar graph on the right. The percentage of proliferating cells/field is the ratio of the nuclear PCNA-positive cells to the total number of nuclei in the field. Scale bar: 200 μm. Corresponding quantifications are presented in the bar graph on the right. Data represent the mean ± SD of four fields per section, four sections per tumor, and two tumors per group. Statistical analysis was performed by Student’s *t* test. ** *p* < 0.01.

To understand the mechanism responsible for the reduction in B16 melanoma growth by rTSR1, we further investigated paraffin-embedded tumor tissue sections for tumor angiogenesis, tumor cell proliferation, and apoptosis by immunofluorescent staining. Tumor microvessels were significantly reduced in rTSR1-treated mice, as revealed by endomucin staining, a biomarker for the luminal side of endothelial cells (Figure 3A). A much-increased level of apoptotic cells was also observed in the rTSR1 treated group, as revealed by terminal deoxynucleotidyl transferase (dUTP) nick end labeling TUNEL staining (Figure 3B). In contrast, proliferative cells were significantly reduced in the rTSR1 treated group compared to the control group (Figure 3C). However, the rTSR1 treatment of B16F10 melanoma cells in the culture did not trigger any apoptosis (Supplementary Figure S1). Hence, the rTSR1 most likely inhibited tumor growth in mice through its suppression of tumor angiogenesis, which then indirectly led to the induction of apoptosis in the tumor cells and the reduction of tumor cell proliferation (Figure 2 and Figure 3). 

**Figure 4:**
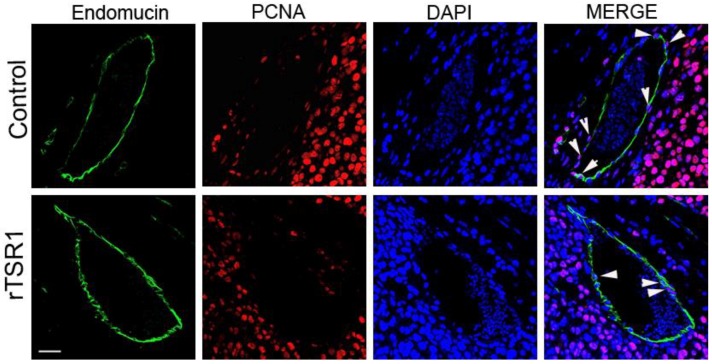
The rTSR1 suppressed tumor endothelial cell proliferation. Representative microscopic images show decreased proliferating endothelial cells in an rTSR1-treated tumor compared to the control. Green indicates the endothelial cells (endomucin-positive), and red indicates the nuclear PCNA-positive proliferating cells. DAPI (4′,6-diamidino-2-phenylindole) was used as the nuclear counter stain. The arrow heads indicate the nuclear PCNA-positive tumor endothelial cells. Scale bar: 30 μm.

**Figure 5:**
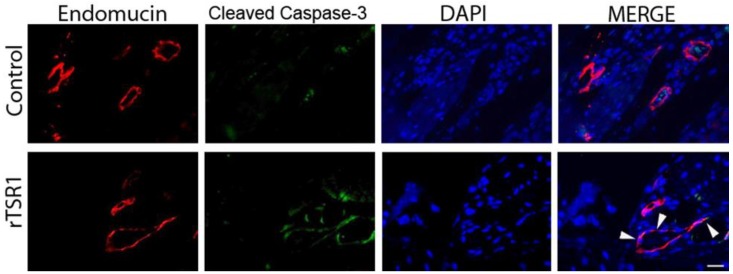
The rTSR1 induced apoptosis in tumor endothelial cells. Representative microscopic images show the increased apoptotic endothelial cells in an rTSR1-treated tumor compared to the control. Red indicates the endothelial cells (endomucin-positive) and green indicates the cleaved caspase-3-positive cells. DAPI was used as the nuclear counter stain. The arrow heads indicates the cleaved caspase-3-positive endothelial cells. Scale bar: 20 μm.

To confirm the anti-angiogenic mechanism of rTSR1 in mouse tumor suppression, we performed double immunostainings of endomucin/PCNA and endomucin/cleaved caspase-3 to detect proliferating and apoptotic tumor endothelial cells, respectively. As shown in Figure 4, there is an obvious reduction in the endomucin/PCNA (nuclear) double-positive endothelial cells facing the lumen of a blood vessel. On the other hand, cleaved caspase-3-positive endothelial cells (endomucin/cleaved caspse-3 double-positive) can be observed in the rTSR1 treated tumors, while this kind of double positive cells could not be found in the untreated control tumors (Figure 5).

Taken together, our results indicate that increased apoptosis (both tumor cells and tumor endothelial cells) with reduced proliferation (both tumor cells and tumor endothelial cells) resulting from a diminished tumor angiogenesis are likely responsible for the decreased tumor mass in rTSR1-treated mice.

**Figure 6:**
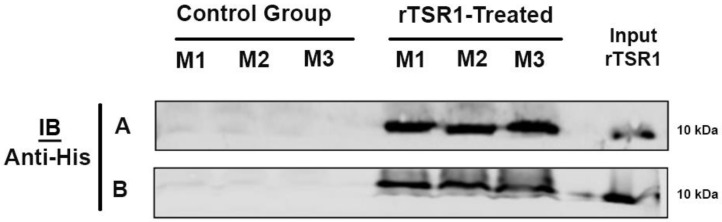
IP-injected rTSR1 reached the tumor. Immunoblot of serum and tumor lysate displayed band at ~10 kDa, corresponding to the molecular weight of rTSR1. The band is observed both in serum (**A**) and with the whole tumor lysate (**B**) from mice that received rTSR1 only. The rTSR1 input is used as the positive control. M1 to M3 refer to mouse 1 to mouse 3.

The reduction of tumor size is linked with the presence of rTSR1 in both the blood circulation and tumor tissues at 15 min post-IP injection of rTSR1 (Figure 6A,B). These results demonstrate that IP delivery of rTSR1 leads to efficient distribution of rTSR1 to the blood circulation, and that a large amount of rTSR1 is already present in the tumor tissue at 15 min after the IP injection. The rTSR1 within the tumor is most likely responsible for the diminished tumor angiogenesis, with a significantly higher level of apoptosis and much less tumor cell proliferation. These factors likely all contributed to the reduced tumor growth observed. 

## 3. Discussion

In this work, we demonstrate that systemically delivered rTSR1 of ADAMTS5 via IP injection potently suppressed distant subcutaneous melanoma growth in mice. This drug delivery approach is clinically applicable for human cancer treatment. We show that the IP-delivered rTSR1 is already present in large amounts in the tumor tissues at 15 min post-peptide injection. These results indicate that rTSR1 can potentially be further developed into an anticancer drug. 

Since the rTSR1 polypeptide is only 75 residues long, it can in principle also be chemically synthesized. The pro-apoptotic activity of rTSR1 is stable between the different batches of the recombinant polypeptides produced. Since bacteria-produced rTSR1 contains no post-translational modifications, and the peptide is produced from insoluble inclusion bodies, the peptide sequence, as well as its spontaneously-formed tertiary structures after purification, is presumed to play a critical role for its anticancer and pro-apoptotic functions. 

Angiogenesis inhibitor proteins and polypeptides are reported to have minimum adverse effect on the quiescent vessel of a healthy individual, but they specifically inhibit pathological angiogenesis. This is a property that makes them as attractive candidates for anticancer drugs [15]. These anti-angiogenic proteins/polypeptides provide a source for designing and developing novel anti-angiogenic agents. As new endogenous angiogenesis inhibitors are continuously being discovered, novel anticancer agents are promising [16]. Thrombospondin-1 (TSP-1) is the first endogenous angiogenesis inhibitor protein identified, and its TSR domains in recombinant forms have been shown to have anticancer activity in mice [17]. Several members of the human ADAMTS family have been reported to have anti-angiogenic and anti-tumorigenic functions [18]. However, not all TSRs are anti-angiogenic, and unique sequences and structures are likely the determinants for a TSR to harbor anti-angiogenic functions. 

While HUVECs underwent apoptosis upon rTSR1 treatment, cancer cells, both B16F10 melanoma and 4T1 breast cancer cells, were found to be insensitive to rTSR1 under cell culture conditions (Figure S1 and data not shown, respectively). Hence, rTSR1 most likely triggered tumor cell apoptosis in mice through its anti-angiogenic activity, which indirectly led to the induction of apoptosis in tumor cells (Figure 2 and Figure 3). Similarly, the suppression of tumor cell proliferation under rTSR1 treatment in mice is likely an indirect effect of tumor angiogenesis inhibition. Previously, Miao et al. reported that the recombinant three TSR domains of human TSP-1 (3TSR/hTSP-1) produced from *Drosophila* S2 cells inhibited tumor growth by suppressing tumor angiogenesis, with the concurrent observation of increased tumor cell apoptosis and reduced tumor cell proliferation [17]. Hence, ADAMTS5 TSR1 produced from *E. coli* without any post-translational modifications seems to have a similar impact on tumors in mice, although the underlining molecular mechanisms and signaling pathways involved may not be the same. 

The fact that the IP-delivered rTSR1 is detectable in serum and tumor lysate at 15 min post-injection suggests a fast and efficient distribution of this polypeptide into tumors. However, the in-vivo stability of rTSR1 is unclear at this stage. Nevertheless, the injection frequency of once every two days was sufficient to inhibit B16F10 tumor growth in mice. 

## 4. Materials and Methods 

### 4.1. Recombinant Type 1 Repeat Domain Production and Purification

The TSR1 domain of human ADAMTS5 corresponds to amino acid residues 567–622. The cDNA sequence corresponding to residues 561–634, which include the surrounding sequences on both the N- and C-terminus of TSR1, were codon-optimized for *E. coli* expression and cloned into a pET30a vector with a N-terminal His-tag (GenScript, Piscataway, NJ, USA). The plasmid construct was transformed into *E. coli* BL21 (DE3) under kanamycin selection. Auto-induction media was used to express rTSR1, and proteins were extracted from insoluble fraction using TRIS buffer containing 8 M urea and with a pH of 8.0. Purification was proceeded using a one-step Ni-NTA column. Fractions were pooled, concentrated, dialyzed, and analyzed by SDS-PAGE and Western blot. Protein concentration was determined with a Bradford protein assay. 

### 4.2. Cell Culture

Human Umbilical Vein Endothelial Cells (HUVECs) were purchased from PromoCell (Cat # C-12200, Heidelberg, Germany), and cultured in EndoGRO-LS complete medium (Millipore, Millipore Corporation, Billerica, MA, USA) on 0.2% gelatin-coated culture flasks at 37 °C in a humidified incubator with 5% CO_2_ (Thermo Scientific, Singapore). The HUVECs of passages three to seven were used for the experiments. B16F10 melanoma cells and 4T1 breast cancer cells were cultured in Dulbecco’s modified eagle medium (DMEM) -high glucose (GE Life sciences, Pittsburgh, PA, USA)) with 10% fetal bovine serum. 

### 4.3. Apoptosis Determination by IncuCyte ZOOM Live-Cell Analysis System

HUVECs were plated in 96-well, flat, clear-bottom, tissue culture grade microplates at the cell density of 5 × 10^3^ cells/well. The following day, the cells were starved in basal media with 2% FBS for 4 h prior to the experiment. Fluorescent conjugated caspase 3/7 substrate (Cat # 4440, Essen Biosciences Ltd., Ann Arbor, MI, USA) was reconstituted in media at the final concentration of 5 μM. Apoptosis was monitored with IncuCyte ZOOM instrument (Essen Biosciences), which was maintained inside a humidified incubator with 5% CO_2_ at 37 °C. Every 1 h for 24 h, phase contrast and green fluorescence channel images were collected from each well. Each well was divided into four quadrants, and every quadrant was imaged using a 10× objective lens and analyzed using IncuCyte ZOOM software, version 2016A. The pan-caspase inhibitor Z-VAD-FMK is from R&D systems (Cat # FMK001, Minneapolis, MN, USA). 

### 4.4. Western Blot

Mice serum and excised tumor lysate were subjected to Western blot analysis. The total protein of 10 μg/lane was resolved with 15% SDS-PAGE and transferred to a nitrocellulose membrane (Cat # 170-4270, Bio-Rad, Philadelphia, PA, USA) using the Trans-Blot Turbo^TM^ Transfer system (Bio-Rad). The membrane was blocked with 3% skimmed milk for 1 h at room temperature. This was followed by overnight incubation with an anti-His antibody (Cat # A00186-100, GenScript, Piscataway, NJ, USA), probing with the respective fluorescent tagged secondary antibody (Cat # 926-32212, LICOR Inc., Lincoln, NE, USA), and visualization using a fluorescent imaging system (Odyssey CLx, LI-COR Inc.).

### 4.5. Immunohistochemistry

Paraffin-embedded tissue sections of 5 μm thickness were de-paraffinized by heating at 60 °C for 15 min followed by Histochoice clearing agent (Cat # H2779, Sigma, St. Louis, MO, USA) for two changes, 5 min each. Then tissues were rehydrated in a series of alcohol (100%, 90%, 80%, and 70% ethanol in double-distilled water) followed by PBS for 5 min. Using 2100-Retriever (Electron Microscopy Sciences, Hatfield, PA, USA), antigen retrieval was performed in sodium citrate buffer (pH 6.0), and then sections were blocked using 3% bovine serum albumin for 1 h in a humidified chamber at room temperature, followed by incubation with anti-endomucin (Cat # SC65495, Santa Cruz, CA, USA) anti-PCNA (Cat # 10205-2-AP, Proteintech, Rosemont, IL, USA), or cleaved caspase-3 (Cat # 9661S, Cell Signaling, Danvers, MA, USA) antibodies at 4 °C overnight. For double staining, tumor sections were incubated with two antibodies together. The next day, the non-specifically bound antibody was washed with 0.1% tween20 in PBS and treated with the respective Alexa fluor 546/488 (Cat # A21206/A11081, Invitrogen, Rockford, IL, USA) for 2 h at room temperature. Apoptotic cells were stained using an in-situ cell death detection kit (Roche, Cat # 11684795910, Basel, Switzerland), based on the TUNEL (TUNEL). The nucleus was counter-stained with DAPI. Fluorescence images were captured using a fluorescence microscope (Zeiss AxioImager M2, Oberkochen, Germany). 

### 4.6. Xenograft Mouse Tumorigenesis Assay

Adult (7- to 8-week-old) female C57BL/6J mice were used in this study. Institution animal care and use committee approval was followed for animal care and experimentation (IACUC; protocol # R16-032, Singapore). Briefly, on day 0, B16F10 mouse melanoma cells (5 × 10^5^) in 100 µL of PBS were injected into the dorsal flank of the mouse. Mice in the control and rTSR1-treated groups were intra-peritoneally (IP) injected with buffer and 200 µg rTSR1/mouse respectively, on alternate days from day 0. Buffers and rTSR1 were filter-sterilized using 0.22 µm filters prior to injection. All mice were monitored daily for their health status, and visible tumor dimensions were measured from seven days post-injection, using a digital caliper, until day 15 post-injection. Tumor volume was determined using the formula Volume = [width^2^ (W^2^) × length (L)/2] mm^3^, where the length is the largest dimension of the tumor and width is the perpendicular dimension. The mice were euthanized using CO_2_ asphyxiation, and blood samples were drawn for serum isolation. Tumor xenografts were surgically excised, with half of the excised tumors used for immunoblot analysis and the other half fixed in 10% formalin and used for paraffin blocking and sectioning.

### 4.7. Mice Serum Isolation 

For serum isolation, 500 µL of blood was drawn from mice through cardiac puncture 15 min after rTSR1 IP injections. From each group (control and rTSR1 treated), blood was collected from three different mice and allowed to clot while standing for 2 h, and serum was separated by centrifugation and then subjected to Western blot analysis. 

## 5. Conclusions

In this work, we presented data indicating that rTSR1 of ADAMTS5 functions as an effective anticancer therapeutic in mice. The tumor suppressive activity of rTSR1 correlated with its inhibition of tumor angiogenesis in vivo and induction of HUVEC apoptosis in vitro. Future studies to understand the molecular mechanisms of its endothelial specific pro-apoptotic activity and additional optimization and modification of the 75 residue polypeptide will help to develop rTSR1 for clinical applications in human cancer.

## Supplementary Materials

The following are available online at http://www.mdpi.com/2072-6694/10/6/192/s1, Figure S1: rTSR1 did not induce apoptosis in B16 melanoma cells.
