# Supplementary material for: Recombinant TSR1 of ADAMTS5 Suppresses Melanoma Growth in Mice via an Anti-angiogenic Mechanism
Source: Cancers (Basel). 2018 Jun 11;10(6):192. doi: 10.3390/cancers10060192 (PMC6025205; doi:10.3390/cancers10060192)
Supplement: Supplementary file 1 [file cancers-10-00192-s001.pdf]

# Recombinant TSR1 of ADAMTS5 Suppresses Melanoma Growth in Mice via an Anti-angiogenic Mechanism

**Bhuvanasundar Renganathan, Vinoth Durairaj, Dogan Can Kirman, Paa Kow A. Esubonteng, Swee Kim Ang and Ruowen Ge**

Supplementary Material

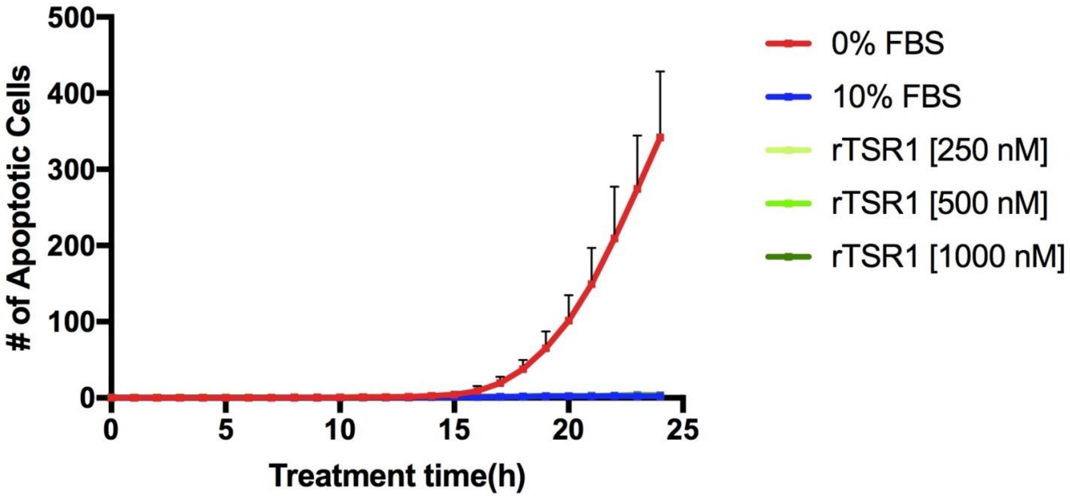


**Figure S1.** rTSR1 did not induce apoptosis in B16 melanoma cells. Various concentrations of rTSR1 (250, 500 and 1000 nM) did not induce apoptosis in B16 melanoma cells. Cells received media with 2% serum served as positive control and cells received media with 10% served as the negative control. Data represents the mean of triplicates ± S.D.
